# The prognostic value of De Ritis (AST/ALT) ratio in patients after surgery for urothelial carcinoma: a systematic review and meta-analysis

**DOI:** 10.1186/s12935-020-1125-2

**Published:** 2020-02-03

**Authors:** Xu Hu, Wei-Xiao Yang, Yan Wang, Yan-Xiang Shao, San-Chao Xiong, Xiang Li

**Affiliations:** 1 West China School of Medicine/West China Hospital, Sichuan University, Chengdu, 610041 People’s Republic of China; 2 Department of Urology, West China Hospital, West China Medical School, Sichuan University, 37 Guoxue Street, Chengdu, 610041 People’s Republic of China

**Keywords:** De Ritis ratio, Urothelial carcinoma, Surgery, Prognosis, Meta-analysis

## Abstract

**Background:**

Recently, the De Ritis (AST/ALT) ratio has been considered as a prognostic biomarker for various malignancies. We conducted this systematic review and meta-analysis to explore the prognostic value of preoperative De Ritis ratio in patients after surgery for urothelial carcinoma.

**Methods:**

We searched the online database Embase, PubMed and Cochrane Library up to October 2019. The hazard ratio (HR) and 95% confidence interval (CI) were extracted from the studies.

**Results:**

A total of 8 studies incorporating 3949 patients were included in the quantitative synthesis. We observed that elevated preoperative De Ritis ratio is associated with inferior OS (HR = 1.97; 95% CI 1.70–2.28; P < 0.001), CSS (HR = 2.40; 95% CI 2.02–2.86; P < 0.001), RFS (HR = 1.31; 95% CI 1.11–1.54; P = 0.001), PFS (HR = 2.07; 95% CI 1.68–2.56; P < 0.001) and MFS (HR = 2.39; 95% CI 1.16–4.91; P = 0.018). Stratified by diseases, the elevated De Ritis ratio also served as an unfavorable factor.

**Conclusion:**

The elevated preoperative De Ritis ratio is an unfavorable factor for patients with urothelial carcinoma. In patients with BC and UTUC, the elevated preoperative De Ritis ratio is also associated with poor prognosis. But De Ritis ratio must be validated in large, independent cohorts before it can be applied widely.

## Background

Urothelial carcinoma (UC) is one of the most commonly diagnosed malignancies, with an estimated 80 thousand new cases and 18 thousand deaths in the United States in 2019 [1]. UC is mainly composed of upper tract urothelial carcinoma (UTUC) and bladder cancer (BC), which are situated at the upper and lower urinary tract, respectively [2]. UTUC is a rare disease that accounts for approximately 5–10% UC, while BC takes up 90–95% of UC and is the most common urinary tract malignancy [3, 4]. Although radical nephroureterectomy (RUN) with bladder cuff excision is the standard treatment for patients with non-metastatic UTUC, high incidences of postoperative disease recurrence have been reported [2, 5]. Radical cystectomy (RC) is the standard approach in patients with non-metastatic muscle-invasive bladder cancer (MIBC) and high-risk non-muscle-invasive bladder cancer (NMIBC), while the 5-year overall survival (OS) rate after RC is approximately 60% [4, 6, 7].

Thus, it is important to precisely predict clinical course after surgery during counseling to determine the suitable treatment and follow-up strategies for individual patients with UC. Pathological T stage and tumor grade are established prognostic factors, besides several prognostic favors are also presented, including lymphovascular invasion, tumor necrosis, Eastern Cooperative Oncology Group Performance Status (ECOG-PS), systemic inflammation and others [2, 4, 6–8].

Aminotransaminases, including aspartate aminotransaminase (AST) and alanine aminotransaminase (ALT), are enzymes released from the liver cell into the blood stream, reflecting hepatocellular damage [9]. The ratio of the serum activities of AST to ALT, firstly described by De Ritis and known as the De Ritis ratio [10]. The De Ritis ratio has been used as a predictor of several chronic liver diseases [11]. Recently, this ratio has been considered as a prognostic biomarker for various malignancies, such as renal cell carcinoma, pancreatic cancer, and breast cancer [12–14]. While in patients with surgically treated UC, the prognostic value of De Ritis ratio is still unclear. Nishikawa et al. found that elevated De Ritis ratio is associated with recurrence-free survival (RFS) in patients with UC, while the other study did not detect the significant association between elevated De Ritis ratio and RFS [15, 16]. As a result, we conducted this systematic review and meta-analysis to explore the prognostic value of preoperative De Ritis ratio in patients after surgery for UC.

## Method

### Literature search strategy

Based on the Preferred Reporting Items for Systematic Reviews and Meta-Analyses (PRISMA) Statement, we conducted this systematic review and meta-analysis. We searched the PubMed, Embase, the Cochrane library up to October 2019. We applied the following items: urothelial carcinoma (urothelial, bladder, tumor, cancer or carcinoma) and De Ritis ratio (aspartate aminotransferase, AST, alanine aminotransferase, ALT, AST/ALT ratio, AST to ALT ratio) as keywords or Mesh. We also screened the reference lists of all eligible studies to ensure comprehensive search. Two reviewers screened the literature independently, any disagreements were resolved by discussing or consulting another one.

### Inclusion and exclusion criteria

We included articles conforming to the following inclusion criteria: (1) random-controlled studies or observational studies; (2) patients were diagnosed urothelial carcinoma and underwent surgery; (3) De Ritis ratio was obtained before surgery; (4) evaluated the prognostic value of preoperative De Ritis ratio, (5) reported available data for analyses, for example: overall survival (OS), cancer-specific survival (CSS), recurrence-free survival (RFS), progression-free survival (PFS) or metastasis-free survival (MFS). The following studies were excluded: (1) non-English language; (2) patients did not undergo surgery; (3) did not involve the De Ritis ratio, (4) no available data for analyses. We did not include conference abstracts owing to incomplete information. Regarding duplicated records, we only included the most recent and informative study.

### Data extraction and quality assessment

Two reviewers extracted items from all eligible studies independently, which are as follows: the name of the first author and published year, enrollment data and location, study type, diseases, intervention, number of patients, age, the cutoff value of the De Ritis ratio, the duration of follow-up. Concerning the clinical outcome such as OS, CSS, RFS, and PFS, we extracted hazard ratio (HR) and 95% confidence interval (CI) from the studies. If the HRs and 95% CI were not revealed, we could calculate the HR and 95% CI based on the method by Tierney [17]. We used the Newcastle–Ottawa Quality Assessment Scale (NOS) to evaluate the quality of the observational studies. And studies with a score of no less than 7 were considered as good quality.

### Statistical analysis

We conducted all statistical analyses by using STATA version 12 (StataCorp, College Station, TX, USA). As for the clinical outcome, we pooled the HRs and 95% CI. And we used Q and I^2^ statistics to evaluate the heterogeneity among studies. A random-effect model was used when we observed the significant heterogeneity (P < 0.10 or I^2^ > 50%); otherwise, a fixed-effect model was used [18]. We also carried out subgroup analyses based on available data. In addition, we performed sensitivity analyses to test the robustness of the final results. In terms of the publication bias, we used Egger’s test and Begg’s test. A two-sided P-value < 0.05 was considered as a statistical difference.

## Results

### Study search

**Fig. 1:**
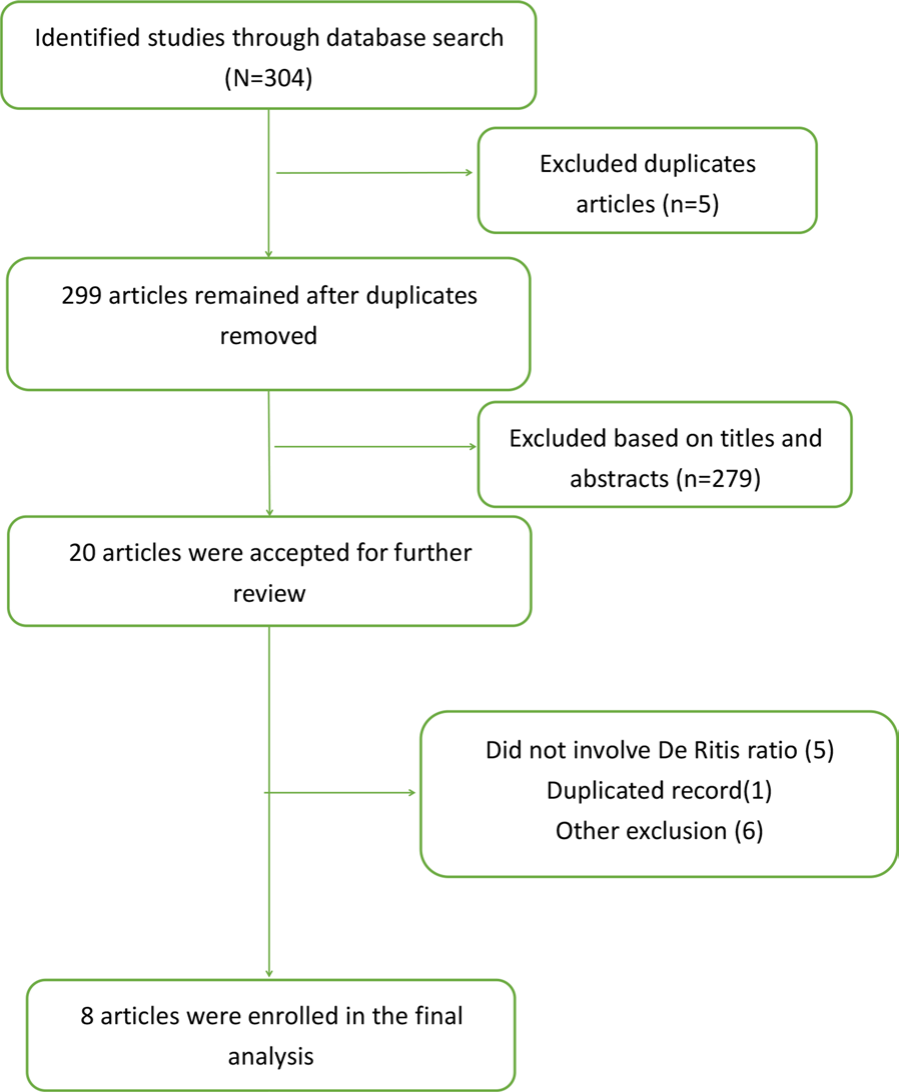
Flow chart of study selection

The study search strategy yield 304 studies, 5 of which were duplicated records. After screening titles and abstracts of the remaining 299 studies, 20 studies were reviewed comprehensively. Finally, a total of 8 studies incorporating 3949 patients were included in the quantitative synthesis [15, 16, 19–24]. The flow diagram of the study search and selection is presented in Fig. 1.

### Characteristics of included studies

Of the enrolled 8 studies, all are published in recent 3 years and are retrospective. Five studies involved the patients with UTUC and underwent RUN [15, 16, 20], 3 studies involved patients with BC and treated by RC [19, 21, 22]. The median ages of patients were similar, ranging from 61 to 69. All studies defined the De Ritis ratio as a dichotomous variable, which was measured before surgery. Most studies had a relatively long follow-up, and median follow-up ranged from 33.3 months to 84 months, while one study did not report the duration of the follow-up [22]. Seven studies observed the OS and CSS, 3 studies revealed the clinical outcome of RFS and PFS, and only one study involves the MFS. Detailed characteristics of the included studies were shown in Table 1. All studies included in our meta-analysis were considered as high quality (Table 1).

**Table 1:** Clinical characteristics of enrolled studies

Study/published year	Enrollment date/location	Study type	Tumor	Treatment	Number of patients	Age (years)	Cut off of AST/ALT	Outcomes	Follow-up (months)	NOS
Yuk 2019 [19]	1991 and 2015/South Korea	Retrospective	Non-metastatic urothelial BC	RC	771	Mean ± SD 64.8 ± 10.2	1.1	OS CSS RFS	Median (IQR) 84 (36–275)	8
Li 2019 [20]	March 1999 and January 2015/China	Retrospective	Localized UTUC	RNU	885	Mean ± SD 66.98 ± 10.55	1.23	OS CSS PFS	Median (IQR) 61.0(38–102)	8
Ha 2019 [21]	August 2008 and May 2013/South Korea	Retrospective	Non-metastatic urothelial BC	RC	118	Median (IQR) 69 (60–74)	1.3	OS CSS MFS	Median 34.1	7
Nishikawa 2018 [15]	2005 and 2015/Japan	Retrospective	Localized UTUC	RUN	135	Median (Range) 69 (52–86)	1.3	RFS	Median 36.1	8
Gorgel 2017 [22]	February 2006 and December 2016/Turkey	Retrospective	Non-metastatic urothelial BC	RC	149	Mean (SD) 61.65 ± 9.13	1.3	OS CSS	NA	7
Cho 2017 [24]	2004 and 2015/South Korea	Retrospective	Non-metastatic UTUC	RUN	1049	Median (IQR) 68.5 (60.5–74.3)	1.6	OS CSS RFS	Median (IQR) 40 (18.4–64.8)	7
Lee 2017[16]	September 1994 and December 2013/South Korea	Retrospective	Non-metastatic UTUC	RUN	583	Median (IQR) 65 (56–72)	1.5	OS CSS PFS	Mean (IQR) 35.0 (16–66)	8
Gao 2017 [23]	March 2005 and August 2015/China	Retrospective	Non-metastatic UTUC	RUN	259	Mean (SD) 67.53 ± 10.43	1.3	OS CSS PFS	Median (Range) 33.3 (15.5–64.2)	7

*UTUC* upper tract urothelial carcinoma, *BC* bladder cancer, *RUN* radical nephroureterectomy, *RC* radical cystectomy, *IQR* interquartile range, *SD* standard derivation, *OS* overall survival, *CSS* cancer-specific survival, *PFS* progression-free SURVIVAL, *RFS* recurrence-free survival, *MFS* metastasis-free survival, *NA* not available, *NOS* newcastle-ottawa quality assessment scale

### Overall survival

**Fig. 2:**
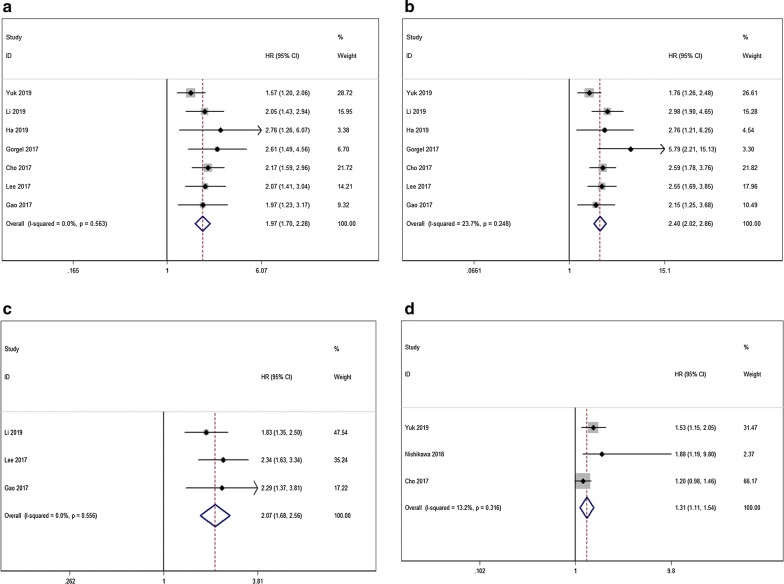
**a** The association between preoperative De Ritis ratio and OS in patients with UC. **b** The association between preoperative De Ritis ratio and CSS in patients with UC. **c** The association between preoperative De Ritis ratio and PFS in patients with UC. **d** The association between preoperative De Ritis ratio and RFS in patients with UC

Regarding 7 studies including 3814 patients, the pooled results demonstrated that elevated De Ritis ratio was significantly associated with worse OS, the pooled HR was 1.97 (95% CI 1.70–2.28; P < 0.001). We did not detect significant heterogeneity among studies (I^2^ = 0.0%, P = 0.563, Fig. 2a).

### Cancer-specific survival

**Fig. 3:**
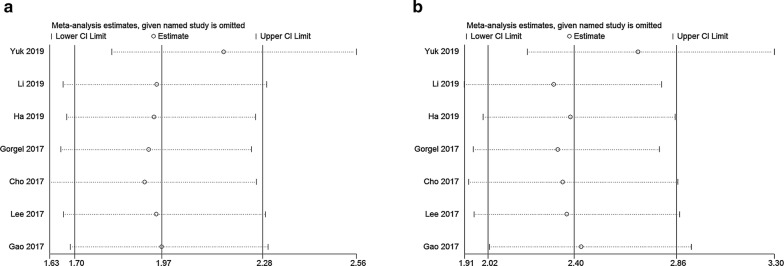
Sensitivity analyses for **a** OS. **b** CSS

As shown in Fig. 3, we demonstrated that the patients with elevated preoperative De Ritis ratio had an inferior CSS. The pooled HR was 2.40 (95% CI 2.02–2.86; P < 0.001). No evidence of heterogeneity was revealed (I^2^ = 23.7%, P = 0.248, Fig. 2b).

### Progression-free survival and recurrence-free survival and metastasis-free survival

In terms of 2 studies incorporating 1727 patients and 1955 patients, we found that the patients with a higher De Ritis ratio had a significantly increased risk of progression and recurrence compared with those with lower De Ritis ratio. The pooled HRs for PFS and RFS were 2.07 (95% CI 1.68–2.56; P < 0.001; Fig. 2c) and 1.31 (95% CI 1.11–1.54; P = 0.001; Fig. 2d), respectively. There was also no heterogeneity among studies. Only one study reported the MFS and higher De Ritis ratio was associated with an increased risk of metastasis (HR = 2.39; 95% CI 1.16–4.91; P = 0.018).

### Publication bias and sensitivity analysis

Because of the small number of included studies, we only performed publication bias and sensitivity analysis for OS and CSS. We carried out sensitivity analyses by removing each study sequentially. After removing each study sequentially, we did not observe a relative change, which showed the stability of our results (Fig. 3). Regarding publication bias, we did not detect significant publication bias of OS and CSS according to the Begg’s test (OS: P = 0.230; CSS: P = 0.230) and Egger’s test (OS: P = 0.059; CSS: P = 0.106).

### Subgroup analyses

Due to the small number of enrolled studies, we only performed subgroup analyses for OS and CSS and stratified by, the number of patients, cutoff value and diseases. For the studies that included ≤ 500 patients, the elevated De Ritis ratio was significant associated with worse OS (HR = 2.30; 95% CI 1.66–3.20; P < 0.001) and CSS (HR = 2.73; 95% CI 1.82–4.10; P < 0.001). And in the > 500 patients subgroup, higher De Ritis was also considered as an unfavorable factor for OS (HR = 1.90; 95% CI 1.61–2.23; P < 0.001) and CSS (HR = 2.34; 95% CI 1.93–2.83; P < 0.001). In cutoff value of ≤ 1.3 subgroup, the patients with elevated De Ritis ratio has a worse OS (HR = 1.88; 95% CI 1.57–2.26; P < 0.001) and CSS (HR = 2.30; 95% CI 1.84–2.88; P < 0.001). Similarly, in the cutoff value of > 1.3 subgroup, higher De Ritis ratio was associated with worse OS (HR = 2.13; 95% CI 1.67–2.71; P < 0.001) and CSS (HR = 2.57; 95% CI 1.95–3.39; P < 0.001). Furthermore, stratified by diseases, elevated De Ritis ratio could serve as an unfavorable factor for OS (HR = 2.08; 95% CI 1.73–2.51; P < 0.001) and CSS (HR = 2.59; 95% CI 2.08–3.21; P < 0.001) in the patients with UTUC. In addition, in the subgroup of BC, the elevated De Ritis ratio was significantly associated with inferior OS (HR = 1.80; 95% CI 1.43–2.27; P < 0.001) and CSS (HR = 2.71; 95% CI 1.38–5.31; P = 0.004). The detailed information was summarized in Table 2.

**Table 2:** Subgroup analyses of OS and CSS

Outcome	Variable	Number of studies	Model	HR (95% CI)	I^2^ (%)	P value of heterogeneity
OS	All	7	Fixed	1.97 (1.70–2.28)	0.0	0.563
No. of patients	≤ 500	3	Fixed	2.30 (1.66–3.20)	0.0	0.670
	> 500	4	Fixed	1.90 (1.61–2.23)	0.0	0.396
Cutoff	≤ 1.3	5	Fixed	1.88 (1.57–2.26)	4.4	0.382
	> 1.3	2	Fixed	2.13 (1.67–2.71)	0.0	0.850
Disease	BC	3	Fixed	1.80 (1.43–2.27)	47.3	0.150
	UTUC	4	Fixed	2.08 (1.73–2.51)	0.0	0.988
CSS	All	4	Fixed	2.61 (2.06–3.31)	5.1	0.367
No. of patients	≤ 500	3	Fixed	2.73 (1.82–4.10)	35.9	0.210
	> 500	4	Fixed	2.34 (1.93–2.83)	30.0	0.232
Cutoff	≤ 1.3	5	Fixed	2.30 (1.84–2.88)	46.5	0.113
	> 1.3	2	Fixed	2.57 (1.95–3.39)	0.0	0.956
Disease	BC	3	Random	2.71 (1.38–5.31)	65.2	0.056
	UTUC	3	Fixed	2.59 (2.08–3.21)	0.0	0.839

*OS* overall survival, *CSS* cancer-specific survival, *BC* bladder cancer, *UTUC* upper tract urothelial carcinoma

– Not available

## Discussion

Despite the development of surgical techniques and adjuvant therapies, the prognosis of patients with UC did not improve a lot. Several prognostic factors were proposed in recent years, including lymphovascular invasion, tumor necrosis, Eastern Cooperative Oncology Group Performance Status (ECOG-PS), systemic inflammation and others [2, 4, 6–8].

In this study, we evaluated the prognostic value of De Ritis ratio in patients with surgically treated UC. We demonstrated that a higher preoperative De Ritis ratio is associated with inferior OS, CSS, PFS, and RFS. When stratified by diseases, the elevated preoperative De Ritis ratio was also an unfavorable factor in patients with either BC or UTUC. Regarding the difference of cutoff values, we divided studies into ≤ 1.3 and > 1.3 groups and found that elevated De Ritis ratio is correlated to poor OS and CSS. In addition, we conducted sensitivity analyses and did not observe a relatively big change. There was also no evidence for publication bias, reflecting the robustness of our results.

So far, several studies have shown the prognostic value of aminotransaminases in patients with malignancies irrespective of the presence of liver-specific disease [12–14, 25, 26]. For example, Stocken et al. found that AST was associated with overall survival in patients with pancreatic cancer [13]. Generally, AST is widely expressed in various tissues such as the brain, muscle, kidney, but ALT is regarded as more liver-specific or enriched [11]. Pathological processes were shown to bring about tissue damage and higher proliferative status, and high tumor cell turnover tends to increase AST rather than ALT, making the De Ritis ratio an attractive potential biomarker [27].

De Ritis ratio, the ratio of the serum activities of AST to ALT, was firstly described by De Ritis [10]. The De Ritis ratio was mostly used as a predictor of several chronic liver diseases in previous studies [11]. Currently, several studies have demonstrated that the De Ritis ratio could serve as a prognostic factor in patients with several cancers. For instance, Bezan et al. enrolled 698 patients with nonmetastatic renal cell carcinoma and found that increased (≥ 1.26) preoperative AST/ALT ratio was an independent prognostic factor for metastasis-free survival (HR 1.61, 95% CI 1.25–2.07, P < 0.001) and OS (HR 1.76, 95% CI 1.34–2.32, P < 0.001). Furthermore, Lee et al. revealed that elevated AST/ALT ratio was an unfavorable factor for OS, CSS, and PFS in patients surgically treated for localized clear-cell RCC [28]. We summarized all available studies and also found that elevated De Ritis ratio is associated with poor prognosis in patients after surgery for urothelial carcinoma.

The association between the De Ritis ratio and histological tumor necrosis, pathological T stage strength this finding. Currently, tumor metabolism has gained attention concerning the carcinogenesis of malignancies. The Warburg effect is the well-known cancer metabolism, describing the abnormal anaerobic glycolysis in cancer cells for producing adenosine triphosphate (ATP) regardless of the availability of the oxygen [29]. Increased glycolysis is shown to be associated with mitochondrial dysfunction linked to nicotinamide adenine dinucleotide (NAD)-related enzymes and glucose transporters [30]. Furthermore, AST is a component of a malate-aspartate shuttle pathway that allows NADH/NAD^+^ conversion [31]. Therefore, the De Ritis ratio might be related to tumor metabolism in many glucose-using cancers. Reportedly, urothelial carcinoma was associated with glucose metabolism [32]. Whyard et al. researched the uptake of fluorescent glucose by bladder cancer cells using fluorescence microscopy and observed significant differences in glucose consumption between normal urothelium and malignant urothelial cells [33]. Based on these findings and our study, it is highly likely that the De Ritis ratio is associated with the prognosis of patients with urothelial carcinoma. But the detailed interaction between the De Ritis ratio and poor prognosis of patients with urothelial carcinoma remains to be explored.

The De Ritis ratio has important implications for clinical practice. The patients with a higher De Ritis ratio had an inferior survival. It may serve as a potential selection criterion for risk factor stratified management of urothelial carcinoma and adjuvant therapies. Besides, close postoperative follow-up should be emphasized for these patients. The AST/ALT ratio is easily accessible and relatively inexpensive because AST and ALT are the most commonly used serum biomarkers in our daily clinical practice.

Despite our novel findings, the present study has some limitations. Firstly, a total of 8 studies incorporating 3949 patients was included in the quantitative synthesis, which is a relatively small number and may limit the power of the final results. So more studies are needed to validate our findings. Secondly, all studies are retrospective, which may increase the risk of bias because of the retrospective data analysis. Thirdly, although included studies tried to exclude all patients with acute or chronic liver disease, they may not eliminate the undetected diseases that confounded results. As results, the De Ritis ratio must be validated in large, independent cohorts before it can be applied widely.

## Conclusion

The preoperative De Ritis ratio is an unfavorable factor for patients with urothelial carcinoma. When stratified by diseases, in patients with BC and UTUC, the De Ritis ratio is also associated with poor prognosis. However, the De Ritis ratio must be validated in large, independent cohorts before it can be applied widely.

## Data Availability

All the data (pooled HR with 95% CI of OS, CSS, PFS, RFS, and MFS) used to support the findings of this study are included within the article.

## Abbreviations

UTUC: Upper tract urothelial carcinoma
BC: Bladder cancer
RUN: Radical nephroureterectomy
RC: Radical cystectomy
OS: Overall survival
CSS: Cancer-specific survival
PFS: Progression-free survival
RFS: Recurrence-free survival
MFS: Metastasis-free survival
NOS: Newcastle–Ottawa quality assessment scale
HR: Hazard ratio
CI: Confidence interval
MIBC: Muscle-invasive bladder cancer
NMIBC: Non-muscle-invasive bladder cancer
ECOG-PS: Eastern cooperative oncology group performance status
AST: Aspartate aminotransaminase
ALT: Alanine aminotransaminase
